# In vitro erythropoiesis: the emerging potential of induced pluripotent stem cells (iPSCs)

**DOI:** 10.1097/BS9.0000000000000215

**Published:** 2024-12-26

**Authors:** Chidera G. Chukwuemeka, Chizaram W. Ndubueze, Adeola V. Kolawole, Joshua N. Joseph, Ifeoluwa H. Oladipo, Ezichi F. Ofoezie, Samuel A. Annor-Yeboah, Abdur-Rahman Eneye Bello, Sodiq O. Ganiyu

**Affiliations:** a Chester Medical School, University of Chester, Exton Park, Chester CH1 4BJ, England; b College of Science, University of Massey, Tennent Drive, Massey University, Palmerston North 4410, New Zealand; c Resilient Agriculture, AgResearch Limited, Grasslands Research Centre Tennent Drive, Fitzherbert Palmerston North 4410, New Zealand; d Department of Biochemistry, Confluence University of Science and Technology, Osara, Kogi State, Nigeria

**Keywords:** Differentiation, Induced pluripotent stem cells, Red blood cells

## Abstract

Due to global blood shortages and restricted donor blood storage, the focus has switched to the in vitro synthesis of red blood cells (RBCs) from induced pluripotent stem cells (iPSCs) as a potential solution. Many processes are required to synthesize RBCs from iPSCs, including the production of iPSCs from human or animal cells, differentiation of iPSCs into hematopoietic stem cells, culturing, and maturation of the hematopoietic stem cells (HSC) to make functional erythrocytes. Previous investigations on the in vitro production of erythrocytes have shown conflicting results. Some studies have demonstrated substantial yields of functional erythrocytes, whereas others have observed low yields of enucleated cells. Before large-scale in vitro RBC production can be achieved, several challenges which have limited its application in the clinic must be overcome. These issues include optimizing differentiation techniques to manufacture vast amounts of functional RBCs, upscaling the manufacturing process, cost-effectiveness, and assuring the production of RBCs with good manufacturing practices (GMP) before they can be used for therapeutic purposes.

## 1. BACKGROUND

Although statistics show that more than 100 million units of blood are donated yearly, there is an increase in the demand for blood.^1^ The available blood banks are insufficient to meet this increased demand,^2,3^ due to a surge in events like armed conflicts, natural disasters, pandemics, and others.^1^ Also, people with chronic diseases like sickle cell anemia depend heavily on transfused blood for their bodies to carry on with oxygen transportation and metabolic activities.^4,5^ Reports show that alloimmunization and blood group incompatibility are the primary reasons why blood transfusion has not been effective in managing these cases.^6^ The incompatibility is due to over 300 blood types (red blood cell [RBC] alloantigens) recorded in humans,^2,7^ making it difficult for the donor population to meet the increasing blood demands. Therefore, it is pertinent that an alternative, safe, and reliable source of blood is made available to make up for these limitations.^8^

Mature red blood cells are produced during erythropoiesis from hematopoietic stem cells (HSC) with the help of the kidney’s erythropoietin (EPO) hormone, growth factors, and cytokines.^9^ HSCs are the starting point for all blood cells. After the division of the HSC, they develop different capabilities and become specialized cells like burst-forming unit (BFU-E), colony-forming unit-erythroid (CFU-E), proerythroblast, early normoblast, intermediate normoblast and late normoblast, reticulocytes, before eventually differentiating terminally into erythrocytes.^10^ RBC production occurs entirely in the bone marrow, comprising extracellular and intracellular factors,^11^ making ex vivo production of erythrocytes difficult.^12^ The mature erythrocytes, also known as RBC, may be obtained from bone marrow or umbilical cord blood (UCB) using various techniques developed over the past few years.^13–15^ However, the same difficulties as voluntary donation remain, such as donor-dependent and lack of diagnostic tools for infectious diseases.^16^ In addition, there are difficulties in acquiring a significant quantity of HSCs for this technique.^17^ It has not been optimized to the level necessary for clinical usage, making it challenging to develop many erythrocytes in vitro from HSC.^18^ Many researchers have worked on developing several alternatives to red blood cells, such as perfluorocarbon emulsions (PFC), liposomes (hemoglobin encapsulation), cell-free hemoglobin with oxygen-carrying potential,^19,20^ but limited vascular circulation which causes damage to the organs, high blood pressure and significant toxicities while used in vivo have proven to be key obstacles to their clinical application.^21,22^

Human pluripotent stem cells (PSCs), such as embryonic stem cells (ESCs) and induced pluripotent stem cells (iPSCs), can multiply endlessly in culture, giving rise to ectoderm, mesoderm, and endoderm lineages.^23–25^ Thus, human PSCs have received much attention as a potential replacement for the present transfusion banking.^26,27^ Human ESCs can differentiate into mature RBCs.^28,29^ However, the use has attracted controversy due to ethical issues.^30^

In recent years, the development of methods for the in vitro production of erythrocytes from iPSCs has shown great potential to overcome the issue of blood shortage with a technological and ethical breakthrough.^1^ The production of iPSCs occurs from adult cells in scientific research, eliminating the ethical challenges of using human embryos.^31^ However, it is vital to note that the production of iPSCs and their usage in research are not entirely devoid of ethical issues. These include an informed consent procedure for acquiring the primary adult cells utilized for reprogramming, ownership, and monetization of iPSC lines.^32^ In general, iPSCs provide a promising solution to ESCs regarding ethical and societal concerns. iPSCs can produce erythrocytes for transfusion support through somatic cell reprogramming.^33^ In 2006, the transcription factors Oct4, Klf4, Sox2, and c-Myc (OKSM), also known as Yamanaka’s factors, when expressed ectopically, enabled the generation of iPSCs from murine fibroblasts, hence enabling the development of a specific therapeutic cell.^34^

Moreover, iPSCs and human ESCs share many similar features, which is why these cells may be an appropriate therapeutic option for the in vitro production of RBCs to solve the problem of blood supply shortages. Bearing this in mind, several researchers over the past 2 decades have tried to generate mature RBCs from iPSCs in vitro.^31,35,36^

Therefore, this review aims to provide a detailed insight into the current state of the art and future research potentials of using iPSCs in the in vitro generation of RBCs.

## 2. iPSC-BASED PRIMITIVE AND DEFINITIVE ERYTHROPOIESIS

Erythropoiesis is the production of mature red blood cells from HSC, and it generally occurs in 2 phases: primitive erythropoiesis and definitive erythropoiesis.^37^ These phases result in RBCs with different functional properties. Primitive erythropoiesis occurs early in gestation, giving rise to transient cells. It generally begins in the developing embryo’s yolk sac, giving rise to large, nucleated erythrocytes expressing embryonic globins such as zeta (ζ) and epsilon (ε).^38,39^ In contrast, definitive RBCs derive from distinct populations of hematopoietic progenitor cells (HPCs), which emerge later in the intra-embryonic arterial system at various embryonic sites.^40,41^ These progenitors eventually migrate to the fetal liver, where they differentiate into erythrocytes that express fetal hemoglobin, are significantly smaller, and possess the capacity to enucleate, ensuring the developing fetus’s oxygen needs are met.^37,42,43^

Studies aiming to generate functional RBCs from iPSCs have demonstrated the capacity to recapitulate these distinct waves of erythropoiesis.^35,44–46^ When directed toward the hematopoietic lineage, iPSCs initially give rise to primitive erythroblasts, resembling the early embryonic wave, which similarly express embryonic globins and are nucleated.^37,47–49^ However, the challenge in generating definitive erythrocytes from iPSCs lies in achieving full terminal maturation, including enucleation and adult hemoglobin expression.^50–52^ Though few studies have reported the transition from primitive to definitive erythropoiesis in murine models,^49,53^ there is still limited information on what exactly drives this definitive wave, as such this has hindered clinical translation.

## 3. TECHNIQUES FOR GENERATION OF RBCs FROM iPSCs

**Figure 1.**
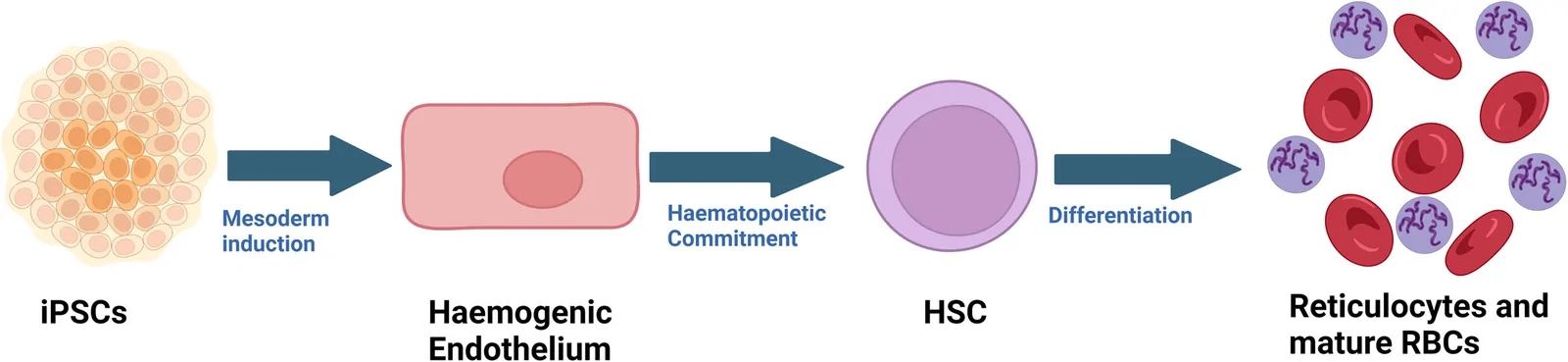
Steps involved in the generation of RBCs from iPSCs. The process of iPSC-based erythropoiesis generally starts from mesoderm induction then proceeds to hematopoietic commitment and finally to erythroid differentiation (maturation of precursors into enucleated RBCs). Figure created with BioRender. HSC = hematopoietic stem cell, iPSC = induced pluripotent stem cells, RBC = red blood cell.

Two methods have generally been used to differentiate iPSCs into RBCs: co-culturing feeder cells with human iPSCs and developing embryoid bodies (EBs) (feeder-free culture).^50^ Differentiating iPSCs into erythrocytes was first done in 2010 using a suspension EB technique.^36^ Other researchers also employed this technique to generate RBCs from iPSCs.^35,47^ However, some other researchers have also employed the use of feeder layer co-cultures.^54–56^ Some reports showed that feeder layer co-cultures yielded better outcomes due to the cellular support.^46,56^ Further research validated this claim as the OP9^56^ and the CH310T1/2^54^ feeder cells have been reported to enhance the generation of RBCs from iPSCs.^55^ However, irrespective of the method used, 3 steps are generally involved in the generation of RBCs from iPSCs viz: mesoderm induction, hematopoietic commitment, and erythroid differentiation (stimulation of erythropoiesis, the proliferation of erythroid precursor cells, and the maturation of precursors into enucleated RBCs) (**Fig. 1**).^36,47,57^ This also involves the use of certain factors, including cytokines (interleukin [IL]-3, IL-6), dexamethasone, stem cell factor (SCF), recombinant human EPO, vascular endothelial growth factors (VEGF), insulin-like growth factor I (IGF-I), Fms like tyrosine kinase 3 (FLT3), bone morphogenetic factor 4 (BMP4), albumin, and transferrin.

Moreover, an ideal technique, as well as a perfect culture environment for human iPSC-derived RBCs, should be able to produce a lot of mature, functioning, and enucleated erythrocytes.^47,56^

Most current regimens have limitations, such as being too complex, costly, time-consuming, and unphysiological because of their high dependence on growth factors and cytokines.^4^ In addition, the steps involved in purifying and digesting the hematopoietic cells further complicate the process and eliminate the possibility of essential cell-to-cell interactions in the artificial microenvironment.^47^ This has resulted in inconsistencies in the yield, efficiency, and maturation of RBC obtained using these procedures.^35,58^

A comparative study of the efficacy of the 2 approaches by Bernecker et al^47^ showed that the enucleation achieved in the feeder layer co-culture system (44% ± 15.1%) was significantly higher (*P* < 0.01) than that of the EB-based suspension culture (20.2% ± 11.7%). This increased enucleation in the feeder layer co-culture might be due to interactions between the erythroid cells and other system components, such as macrophages and stromal components.^56^ Reports by other researchers further substantiated this hypothesis. The co-cultures of erythroblasts and stromal cells injected into NOD-SCID mice showed enhanced enucleation.^59–61^

## 4. POTENTIALS OF iPSC-DERIVED ERYTHROCYTES IN VIVO

Research on the production of RBCs from iPSCs has been extensive, but to date, there have been limited clinical studies involving the transfusion of erythrocytes produced from iPSCs.^2^ The lack of data on the in vivo functioning of iPSC-derived blood cells is a critical hurdle that must be overcome before these cells can be used safely and effectively in therapeutic settings.^62^

The murine mice have been employed to test the effectiveness of both autologous and xenogeneic iPSCs-derived RBCs by suppressing their immune system.^63^ However, the total loss of immunity and the toxicity of the compounds (clodronate liposomes and cobra venom factors) used to achieve immune suppression make murine mice inefficient models for testing the in vivo efficacy of the iPSCs-derived RBCs.^64^

Five healthy donors’ peripheral blood polymorphonuclear cells (PMN) were reprogrammed into iPSCs, which were then effectively differentiated into erythrocytes and implanted into NOD-SCID gamma mice (NSD mice).^49^ Before transplantation, only about 13% of generated erythrocytes achieved enucleation, but 1 day after infusion into the mice models, over 95% of the cells achieved enucleation. This study further showed that as iPSCs-derived erythroblasts mature in culture, they gradually lose the markers of early erythroblasts: CD49d and CD71. However, residuals of these markers remained present on the mature erythroblasts in vitro. After these erythroblasts were transfused into experimental mice, they showed phenotypic transformation and a complete loss of the CD49d and CD71 markers. Instead, they expressed the markers of mature RBCs: CD233 and CD235a. This outcome suggests the possibility of iPSC-derived RBCs undergoing transformational changes and attaining maturity in vivo. Hence, further research needs to be done to validate this possibility. Also, the factors necessary for achieving enucleation and becoming mature RBCs might naturally occur in vivo, including the interaction of transfused cells with bone marrow macrophages.^49^ However, the use of human platelet lysate (hPL), a fibrinogen-depleted supplement prepared by plateletpheresis, which is reported to be richer in growth factors, cytokines, and other proteins than bovine serum,^65,66^ might have also contributed to the success rate recorded in the study. Applying the correct statistical tool (1-way analysis of variance test) in the investigation to compare the data further validated the result. Another similar study by Kobari et al^58^ observed that after sublethally irradiating NOD/SCID mice and transfusing them with iPSC-RBCs, globin switching from fetal to adult hemoglobin occurred in vivo. During iPSC-SCD differentiation and maturation, there was 44% globin alpha, 29% gamma-G, 15% gamma-A and epsilon, and 7% zeta. However, the percentage of enucleation was not analyzed due to the low level of erythrocytes circulating in the mice models. This may be attributed to these researchers’ use of albumin in the culture medium, as it has been previously suggested that albumin does not contain as many factors as hPL.^67^ Moreover, Deng et al^49^ reprogrammed iPSCs from PMN cells, whereas Kobari et al^58^ utilized fibroblasts. The origin of the starting cell population may have also affected the various outcomes from these studies since iPSCs have been shown to retain epigenetic memory.^68–71^

Despite the success of these studies, there were some limitations. Statistical approaches such as power analysis were not employed to determine the sample size. Moreover, the follow-up period of enucleation and globin switching in vivo was short (4 weeks). It cannot be ascertained if the cells will achieve the average life span (90–120) and oxygen-carrying capacity of normal RBCs in the peripheral circulation for longer. Also, the choice of laboratory animals might be a limiting factor. It has been reported that the NSG mice used in these experiments are often associated with aberrant bone marrow, altered cytokine production, and suppressed immune systems, making them unfit for in vivo studies.^72^ The absence of immunity in these mice also leads to poor survival of human iPSCs; therefore, the option of using non-human primates like Baboons (known to have some developmental similarities with humans) for preclinical studies should be explored.^64^ However, before human clinical trials are done, further studies using relevant animal models must be conducted.

## 5. RECENT ADVANCES IN iPSC-BASED RBC PRODUCTION

Improvements in the efficiency and scalability of the differentiation process have been at the forefront of recent developments in the production of iPSC-RBCs.^49^ Poor erythrocyte enucleation, generation of the beta adult form of globin, and a large number of RBCs (10^12^) required to produce a single unit of RBC for transfusion have been the major obstacles preventing the translation of iPSC-RBC into clinical practice.^4^

Numerous studies on advancing erythrocyte differentiation procedures have been conducted in response to this desire for improved efficiency and scalability. Researchers are constantly exploring ways to improve the cell’s efficiency, yield, and maturation by modifying the existing procedure.^73^ Some studies have found many microRNAs (miRNAs) are involved in RBC terminal differentiation and enucleation, and these miRNAs may improve iPSC-RBC yield.^74,75^ For instance, overexpression of erythropoiesis-related miR-451 and miR-144 enhances iPSC differentiation into RBC,^76^ and the inhibition of miR-125b, miR-30A, and miR-93 have been shown to improve the enucleation and yield of mature erythrocytes.^77,78^ In a similar study, lncRNAs that have been shown to inhibit programmed cell death also enhance iPSC-RBC production.^79^ Also, 3D scaffolds, including poly (D, L-lactide-co-glycolide) and porous polyvinyl fluoride resin, stimulate the bone marrow microenvironment and promote erythroid cell survival.^80^

Some other novel studies have also introduced suspension platforms and spinner flasks to improve differentiation, especially as the major challenge associated with the already existing differentiation protocols, particularly the formation of EBs, is the difficulty in differentiation under certain chemically induced culture conditions.^81^ A 2016 study intended to improve differentiation by combining the expansion of undifferentiated iPSCs and the formation of EBs in suspension using spinner flasks, followed by initiating differentiation on a platform rocker.^51^ Though this study did not report any enucleation rate, over 64% of the cells generated expressed adult hemoglobin and exhibited oxygen-carrying capacity. Spinner flasks have also been combined with a suspension culture platform and OP9 stromal cells to differentiate iPSCs into erythrocytes with an enucleation rate ranging from 18.1% to 59%.^52^ The same author also reported an enucleation rate of 28% to 40% with a suspension culture platform together with human mesenchymal stem cells (hMSC) as the feeder layer,^73^ however considering the higher enucleation rate recorded with OP9 cells, these cells may work better with the suspension culture platform than the hMSCs.

Furthermore, a 2019 research also developed 2 culture media (R6 and IMIT) and combined them to differentiate iPSCs and PMNs into erythrocytes.^72^ This improved media lacks albumin and several animal components. Moreover, the media employed significantly less transferrin than previous protocols, and enucleation rates between 40% and 90% were reported. iPSCs reprogrammed from baboon peripheral blood CD34+ cells have also been differentiated into erythrocytes with an enucleation rate of 40% to 50% using the same novel culture conditions.^64^ These findings contrast with those from similar research, where a monolayer-based approach which requires no replating, co-culture or EB formation was used to differentiate iPSCs reprogrammed from peripheral blood CD34+ cells into HPCs. However, the cells generated failed to mature in vivo when transplanted into female NSG mice.^53^ This discrepancy may have been due to the system employed for the differentiation. More so, it is observed that the researchers, in a bid to improve further differentiation, aimed to modulate the wingless-related integration site (WNT)/β-catenin and activin/nodal/transforming growth factor β (TGFβ) signaling pathways by the addition of CHIR/SB molecules during mesodermal specification. Existing evidence in the literature suggests that this alone is insufficient in improving hematopoietic specification and maturation.^43,82,83^ This invariably means that further improvements must be made to the method to ensure absolute efficiency.

## 6. FACTORS AFFECTING iPSC-BASED GENERATION OF ERYTHROCYTES

**Figure 2.**
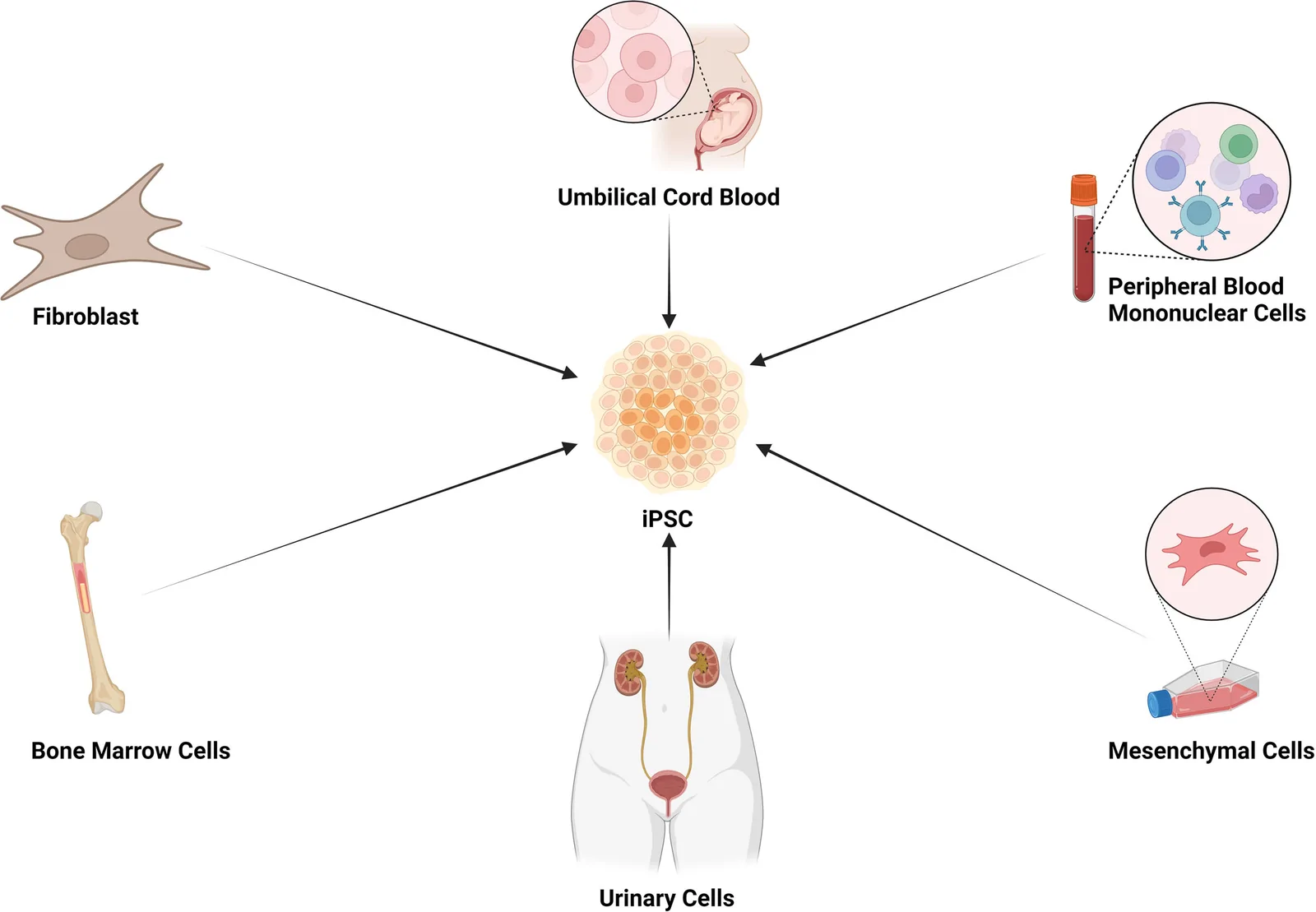
Some of the starting materials (cell sources) for the in vitro generation of RBCs from iPSCs. Figure created with BioRender. iPSC = induced pluripotent stem cells, RBC = red blood cell.

Fibroblasts,^84,85^ cord blood CD34+ cells,^86^ peripheral blood mononuclear cells, mesenchymal stem cells,^48^ human urinary cells,^87^ CD36+ erythroblasts,^47^ and bone marrow stromal cells^54^ have all been used to generate RBCs from iPSCs (**Fig. 2**). However, due to epigenetic memory and control, it has been proposed that the starting cell type might play a crucial role in the efficacy of the process.^35,68,88^ In one of the published studies, MSC-derived iPSCs yielded more definite erythroid cells with elevated beta-globin than those derived from peripheral blood.^48^ In other experiments, UCB CD34+ cell populations with MSCs-like characteristics yielded better erythroid cells than fibroblast-derived cells.^35,84^ The high yield of erythroid cells observed when UCB CD34+ cells are used as starting materials has also been attributed to their high proliferation rate and stemness.^89^ Moreover, the reduced mutation incidence, decreased immunological reactions, and the non-invasive technique used in obtaining these cells make them the preferred starting materials over adult cells.^90^ Therefore, scientists must intensify research to determine which epigenetic signals in these starting materials play critical roles during erythropoiesis.^91^

Aside from the starting materials, factors like the reprogramming vectors/plasmids and culture conditions (serum-free or feeder-free) are also critical in the in vitro generation of RBCs from iPSCs.^44,85^ A minor drug, UM171, has been reported to enhance the in vitro differentiation of iPSCs into erythroid cells.^87,92^ There have also been reports that NADPH oxidase and histone deacetylase enhance the nascent stages of erythroid differentiation from iPSCs^93,94^ and chromatin condensation in the generated erythroid cells, respectively.^95^ Certain miRNAs,^96^ and long noncoding RNAs (lncRNAs) that inhibit apoptosis and promote erythropoiesis,^97^ have also been reported to facilitate the differentiation of iPSCs into RBCs.

## 7. OBSTACLES/LIMITATIONS PREVENTING CLINICAL THERAPEUTIC APPLICATION

Despite the giant strides recorded in attempts to generate RBCs in vitro by differentiating iPSCs, no clinical trials have been conducted.^8^ Human iPSCs may be viewed as a limitless supply of RBCs compared to HSCs. However, generating mature RBCs from iPSCs is still inefficient, necessitating less stringent experiment techniques.^2^ Before the clinical translation of iPSCs-derived RBCs, the process-associated risks and challenges must be eliminated.^98,99^ Some of these issues include safety practices, ethical considerations, the high cost of materials, and others.

Good manufacturing principles (GMP) must be followed during the in vitro generation of RBCs from iPSCs to ensure minimum immunogenic effects from these cells following delivery to patients.^8^ Xenogenic and undefined components (such as feeder cells and bovine serum albumin) used in experiments of this nature can induce immunological responses.^8,44,46,85^ Therefore, for clinicians to proceed with clinical trials, the techniques involved in RBC generation from iPSCs must incorporate a serum-free culture system or chemically defined media.^100^ Also, safety evaluation in animal models is highly encouraged.^2^

The expensive media used in generating RBCs from iPSCs is costly, making the process a bit inefficient in growing RBCs on a large scale.^2^ Also, the modification processes, such as the shift from 2D monolayer cells to scalable 3D bioreactor settings and the use of large amounts of cytokines and growth factors, are all capital-intensive.^8^ Therefore, cost-effective approaches that enhance differentiation and enucleation should be explored.^8,10^

The dependence on the conventional way of generating RBCs from iPSCs in flasks rather than large-scale industrialization makes it difficult to generate sufficient pints of blood.^8^ Currently, researchers are intensifying studies using the 3D culture techniques, which are said to be better than the 2D techniques.^101^ Novel reactor designs, such as the creative vertical-wheel bioreactor, are being explored.^101,102^ High concentrations of many cytokines are also being used to compensate for insufficient knowledge of the molecular events that occur during embryogenesis.^46^ However, to effectively maximize the bioreactor system approach, research must be intensified to understand the nutrient and oxygen requirements and the waste elimination process of the various techniques.^8^

There is also still a poor understanding of exactly how some of the molecules and factors used to induce differentiation operate precisely and if any of them may possess any potential deleterious effect.^103^ Most studies carried out in mice models had relatively short follow-up periods. Since physiological RBCs have a life span of 120 days, it is still unclear if the transplanted cells can maintain a similar lifespan while functioning efficiently.

Poor growth rates, inefficient enucleation,^104^ the number of cells needed to obtain transfusible pints,^4^ inefficient antigen profiling, poor screening for mutations,^105^ and large amount of embryonic and fetal hemoglobins,^5,8^ are all factors limiting the in vitro generation of RBCs from iPSCs. Therefore, further research should be carried out to eliminate these variables.^2^

Despite these limitations, using iPSCs in RBC generation still holds great promise for transfusion medicine. RBCs derived from human iPSCs can be genetically modified through research and used to treat various diseases.^8^ Extracellular vesicles (EVs) are derived from the membrane of iPSCs-generated RBCs and are employed in gene editing and drug delivery, thereby aiding treatment.^106^ These EVs are released during erythroid growth in vitro, while in vivo, complement-mediated calcium influx and vesicle shedding trigger their release from aging RBCs in circulation.^8^ They are used as delivery agents instead of synthetic transfection agents like viruses and nanoparticles.^106^ Some researchers have efficiently delivered long and short RNAs into cancer cells, validating their usefulness in generating novel medicinal products.^107^ Therefore, these EVs from iPSCs-derived RBCs will be of much use in genetic modification/therapies.

## 8. CONCLUSION

iPSCs have become essential in vitro sources of RBCs, and efficient differentiation of iPSCs into RBCs is key for actualizing a potential therapy and solutions to the problems associated with blood transfusion shortages. Unfortunately, several obstacles must be overcome before this method can be extensively used for therapeutic purposes. More research and development are required for quality control, cost-effectiveness, scalability, compatibility with transfusion procedures, and the following of GMPs with well-defined, simple, and robust methods to facilitate its translation to clinical practice. However, with the recent advances in the production of iPSC erythrocytes and the use of engineering and novel techniques such as the perfusion bioreactor system and miRNAs, it is possible to generate large amounts of blood cells required for transfusion. This would represent a significant advancement in transfusion therapy, offering a possible solution to the lack of safe and compatible blood products for needy patients.
